# Natalizumab-Related Progressive Multifocal Leukoencephalopathy in Multiple Sclerosis: Findings from an Italian Independent Registry

**DOI:** 10.1371/journal.pone.0168376

**Published:** 2016-12-20

**Authors:** Luca Prosperini, Nicola de Rossi, Cristina Scarpazza, Lucia Moiola, Mirco Cosottini, Simonetta Gerevini, Ruggero Capra, on behalf of the Italian PML study group

**Affiliations:** 1 Dept. of Neurology and Psychiatry, Sapienza University, Viale Dell’Università, Rome, Italy; 2 Multiple Sclerosis Centre, Spedali Civili di Brescia, Via Ciotti, Montichiari, Brescia, Italy; 3 Dept. of Neurology, San Raffaele Scientific Institute, Via Olgettina, Milan, Italy; 4 Dept. of Translational Research and New Surgical and Medical Technologies, University of Pisa, Via Paradisa, Pisa, Italy; 5 Dept. of Neuroradiology, San Raffaele Scientific Institute, Vita-Salute San Raffaele University, Via Olgettina, Milan, Italy; Heinrich-Heine-Universitat Dusseldorf, GERMANY

## Abstract

**Background:**

The monoclonal antibody natalizumab (NTZ) is a highly effective treatment for patients with multiple sclerosis (MS). However, this drug is associated with increased risk of developing Progressive Multifocal Leukoencephalopathy (PML), an opportunistic infection of central nervous system (CNS) caused by the John Cunningham polyomavirus (JCV).

**Objective:**

To describe the 12-month clinical course of 39 patients with MS (28 women, 11 men) who developed NTZ-related PML after a mean exposure of 39 infusions.

**Methods:**

An Italian independent collaborative repository initiative collected and analyzed socio-demographic, clinical, magnetic resonance imaging (MRI) data and number of JCV-DNA copies detected on cerebrospinal fluid (CSF) samples of patients diagnosed as affected by NTZ-related PML. The evolution of disability, measured by the Expanded Disability Status Scale, was assessed at NTZ start, at PML diagnosis and after 2, 6 and 12 months from PML diagnosis. The effect of clinical and paraclinical characteristics at PML diagnosis on the final outcome was also investigated.

**Results:**

Ten patients (25.6%) were diagnosed before 24 NTZ infusions. In six cases (15.4%) the PML suspect was made on the basis of highly suggestive MRI findings in absence of any detectable change of clinical conditions (asymptomatic PML). In patients with symptomatic PML, the diagnosis was quicker for those who presented with cognitive symptoms (n = 12) rather than for those with other neurological pictures (n = 21) (p = 0.003). Three patients (7.7%) died during the 12-month observation period, resulting in a survival rate of 92.3%. Asymptomatic PML, more localized brain involvement and gadolinium-enhancement detected at MRI, as well as lower viral load were associated with a better disability outcome (p-values<0.01).

**Conclusion:**

Our findings support that early PML diagnosis, limited CNS involvement and initial signs of immune restoration are associated with a better outcome and higher survival rate, and confirm the utility of MRI as a surveillance tool for NTZ-treated patients.

## Introduction

Progressive multifocal leukoencephalopathy (PML) is an opportunistic infection that usually occurs in immunocompromised patients [1]. The causative agent of PML is the John Cunningham virus (JCV), a polyomavirus which mainly infects oligodendrocytes and astrocytes, even if some variants may involve neurons (cortical pyramidal and cerebellar granule cells) [2,3].

In addition to being widely studied in patients with human immunodeficiency virus (HIV) over the last decade [4], PML has also been observed in patients with multiple sclerosis (MS) treated with natalizumab (NTZ) [5–7], a monoclonal antibody which suppresses immune surveillance in the central nervous system (CNS) by preventing the immune system to enter the CNS [8,9]. The estimated incidence of NTZ-related PML is in the order of 4.15 per 1000 patients, with a mortality rate of about 24%, according to manufacturer report [10]. Patients treated with NTZ are considered at higher risk for developing PML if they have tested positive for JCV, and have received more than 24 infusions, and either if they have previoulsy used an immunosuppressant, or have not used immunosuppressants and have a high JCV antibody index [11,12].

The course and outcome of PML is influenced not only by the lytic action of JCV, but also by the impact of the immune reconstitution inflammatory syndrome (PML-IRIS), i.e. the excessive inflammatory reaction occurring as a result of the reconstitution of the immune system in a previously immunocompromised patient [13]. In the event of PML-IRIS, the overwhelming immune response is directly responsible for the worsening of the patient’s clinical condition [14] and the magnetic resonance imaging (MRI) findings of extension of the lesion(s) coupled with enhancement and oedema [15].

Some clinical and paraclinical features at NTZ-related PML diagnosis have been reported to be predictive of bad outcome, including older age, high pre-PML disability, MRI findings consistent with multifocal damage, elevated number of JCV copies detected in cerebrospinal fluid (CSF) and symptomatic PML [16–18]. However, literature data regarding clinical outcomes after PML suffer from several biases, including incomplete data collection, largely heterogeneous management of patients and not univocal definition of PML-IRIS [15, 19]. This latter point is of particular relevance, since a correct interpretation of MRI is crucial to determine appropriate therapeutic strategy. Inflammation detected at MRI does not necessarily imply the occurrence of PML-IRIS. A productive brain reaction against JCV can be also detected as perivascular inflammation and enhancement (inflammatory PML). In this latter case, misdiagnosis as PML-IRIS and consequent treatment with early corticosteroids may lead to the worsening of patient’s clinical outcome [20].

Therefore, in the current paper we would carefully illustrate the clinical characteristics of 40 Italian patients with MS who developed NTZ-induced PML and their longitudinal clinical evolution up to 12 months after the PML diagnosis. In addition, we sought to identify if there was any socio-demographic, clinical, or MRI feature at PML diagnosis that influenced the longer-term outcome.

## Materials and Methods

### Patients ascertainment

An Italian independent spontaneous collaborative repository initiative made a registry for the collection of complications in patients with MS treated with NTZ. Thirty two Italian sites took part to the initiative. Data of patients who received a definite, probable or possible diagnosis of NTZ-related PML according to AAN criteria [21] were retrospectively collected and stored by the MS centre of Montichiari (Brescia), the reference site for NTZ-related PML cases in Italy.

Each site regularly followed-up the patients from the start of NTZ treatment according to standard clinical practice and recommendation provided by the Italian regulatory agency (Agenzia Italiana del Farmaco, AIFA) [22]. Clinical data including Expanded Disability Status Scale (EDSS) [23] scoring and relapse recording were then prospectively collected with at least quarterly evaluations from NTZ start [24]. Patients were scanned for brain MRI with 1.5T or 3.0T magnets at NTZ start and at least yearly during NTZ treatment, according to standardized procedures [25]. Since the introduction of STRATIFY test to detect serum anti-JCV antibodies in 2011 [26], patients with a negative anti-JCV antibody test were re-tested periodically (at least yearly), and those who were at high risk of developing PML were scanned every 3 or 4 months throughout NTZ treatment [5].

CSF samples were collected at PML suspicion and sent to the National Institute of Health (NIH) Laboratory of Molecular Medicine and Neuroscience (Bethesda, MD, USA) in 35 cases; 4 specimens were analyzed in local labs; the remaining specimen was analyzed in FOCUS lab (Cypress, CA, USA). JCV-DNA copies were searched on CSF samples by quantitative polymerase chain reaction (PCR).

The retrospective analysis of patients’ data was approved by the ethical committee of the Spedali Civili of Brescia and was conducted in accordance with specific national laws and the ethical standards laid down in the 1964 Declaration of Helsinki and its later amendments. In no way did this study interfere in the Care received by patients.

### Data collection

Socio-demographic, clinical and MRI data were extracted by patient charts and stored into a centralized database.

Socio-demographic data included: sex; age at NTZ start; age at PML diagnosis.

**Fig 1:**
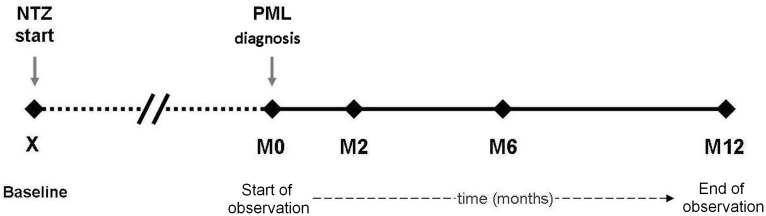
Assessments of patients who developed natalizumab-related progressive multifocal leukoencephalopathy.

Clinical data included: previous immune suppression; total number of NTZ infusions; annual relapse rate (ARR) in the year before NTZ start and during NTZ treatment; symptomatic or asymptomatic PML (i.e. detection of PML lesions at brain MRI scan in absence of new signs or symptoms); number of JCV-DNA copies detected with PCR performed on CSF samples at PML suspect; longitudinal EDSS scores collected at NTZ start (hereafter referred as baseline), at PML diagnosis (M0), at two months (M2), six months (M6) and 12 months (M12) from PML diagnosis (see Fig 1 for the main time frames considered in our patients).

MRI data included: presence or absence of contrast-enhancement in PML lesion(s); presence or absence of the radiological features of PML-IRIS (see below); lesion patterns classified as unilobar (confined to one lobe), multilobar (involving two or more contiguous lobes), widespread (involving two or more non-contiguous lobes and/or present in both hemispheres) [27] and infratentorial. We considered infratentorial PML lesions separately from the other patterns for two main reasons: (i) infratentorial lesions are known to have high predictive value for long-term disability even in MS pathology [28–30]; (ii) infratentorial lesions, particularly if involving the brainstem, need to be considered with particular attention due to the potentially life-threatening complications of oedema and mass effect in these situations [6].

### MRI data harmonization

Brain images were reviewed centrally by the sites of Brescia (Montichiari) and Milano (San Raffaele). Two neurologists (RC, NDR) and two neuroradiologists (MC, SG) reviewed the data independently. Clinical and MRI data were subsequently discussed in parallel to avoid potential bias due to conflicting definition of PML-IRIS [10,20,31–33].

We defined PML-IRIS as the presence of specific radiological changes of PML lesion associated with an almost concomitant unexpected abrupt and severe worsening of clinical conditions [13–15,34]. Radiological features of PML-IRIS consisted in an important inflammatory reaction with swelling or oedema of PML lesion(s) and possible mass effect [29–34]. A “ring-like” or “wave-like” alteration of the blood-brain barrier (BBB) at the periphery of the PML lesion(s) may be also detected [29–34]. We differentiated PML-IRIS from the so-called “inflammatory PML”, whose MRI features are characterized by oedema without significant mass effect, somewhat associated with punctuate BBB disruption not only in PML lesion(s), but also in other parts of the brain [35]. This MRI pattern is associated with slow and steady worsening of clinical status [35], which is thought to be temporally related with recovery of the immune system and inflammatory response to JCV [36].

### Statistical analyses

Categorical variables are expressed as count (percentage) and continuous variables as mean (SD) or median (range), as appropriated. Between-group differences were tested using the Chi-squared or the U Mann-Whitney tests for categorical and continuous variables, respectively. The longitudinal clinical course of patients was explored by repeated measures analysis of the variance (RM-ANOVA) after testing the assumptions of sphericity and normality at each assessment, and with the Student-Newman-Keuls (S-N-K) as post-hoc test. The EDSS score was defined as dependent variable and time (5 levels: baseline, M0, M2, M6 and M12) as the within-subject factor. Potential predictors of clinical course were investigated either by inserting categorical variables of interest as between-group factor or by performing Spearman Rank Correlation coefficients between continuous variables of interest and EDSS changes, calculated by subtracting the EDSS score at baseline from the EDSS score at each of the following assessments (M0, M2, M6, M12) for each patient.

Due to the exploratory nature of this study, no correction for multiple comparisons was applied.

All data were analyzed by using the Statistical Package for Social Science version 16.0 (IMB SPSS, Chicago, Ill, USA).

## Results

### Patient demographic and clinical features

From January 2009 to April 2016, 44 patients with MS received a diagnosis of NTZ-related PML in Italy, and 39 among them were inserted into the database (S1 Table). Their main socio-demographic and clinical characteristics are reported in Table 1.

**Table 1:** Socio-demographic and clinical features of each individual patient (n = 40).

Patients	Age at PML onset (years)	Gender	Disease Duration (years)	ARR pre-natalizumab	ARR during natalizumab	EDSS at NTZ beginning	EDSS at PML onset	Number of infusions
Overall*	41.8 ± 8.5	11 M; 28 F	13.6 ± 7.2	2 (0–4)	0 (0–1)	3.9 ± 1.9	4.8 ± 2.1	38.6 ± 18.2
95% CIs	39.1 to 44.6	7 to 18 M; 22 to 33 F	11.3 to 15.9	1.54 to 2.05	0.03 to 0.23	3.3 to 4.5	4.1 to 5.5	32.7 to 44.5
1	47	F	18	2	0	5.5	6.5	17
2	52	M	12	3	0	2.0	6.0	11
3	38	F	12	2	0	2.0	3.0	21
4	30	F	7	1	0	6.5	8.0	50
5	43	F	2	1	0	3.5	3.5	45
6	46	M	11	2	0	2.0	2.0	30
7	32	F	3	3	0	2.0	4.0	20
8	43	F	N/A	1	0	6.5	7.0	34
9	54	F	22	2	0.05	5.0	6.0	42
10	23	F	11	1	0	2.5	2.0	44
11	51	F	N/A	3	0	6.5	6.5	21
12	46	F	16	2	0	5.5	4.5	39
13	46	M	20	1	0	6.0	7.0	17
14	46	M	8	1	0	3.0	3.5	29
15	51	M	22	1	1	3.5	5.0	34
16	28	F	4	3	1	1.0	3.5	25
17	41	F	20	2	1	6.5	8.5	31
18	60	F	36	2	0	6.0	7.5	13
19	47	F	19	1	0	5.5	7.5	21
20	48	F	14	2	0	3.5	3.5	16
21	56	F	14	2	0	4.0	4.0	48
22	22	F	5	3	0	2.0	2.5	51
23	47	F	24	2	0	7.0	6.5	37
24	43	M	25	1	0	6.5	6.5	63
25	38	M	11	2	0	1.5	2.5	27
26	39	F	10	2	0	2.0	2.0	45
27	45	F	9	1	0	1.5	3.0	45
28	37	M	7	1	0	4.0	6.5	52
29	29	M	6	1	0	2.5	3.0	24
30	38	F	16	1	0.5	4.5	8.0	37
31	36	F	17	2	0.17	3.5	5.5	78
32	46	F	11	4	0	2.0	2.0	64
33	34	F	6	2	1	4.0	5.0	58
34	38	M	18	1	0	3.0	7.5	67
35	42	F	10	1	0	2.5	3.5	28
36	49	M	11	2	0	3.5	3.5	86
37	44	F	21	2	0.3	6.5	7.5	27
38	38	F	17	N/A	0.01	0.0	0.0	55
39	38	F	9	2	0	6.0	3.5	55

* Values are mean ± standard deviation, or median (range) or number.

95% CIs: 95% confidence intervals; F: female; M: male; ARR, annual relapse rate; EDSS, Expanded Disability Status Scale; PML, progressive multifocal leukoencephalopathy; N/A: not available.

Five patients were not included in the database for the following reasons: data unavailability for three patients, including too short observation period; one patient refused to provide informed consent; in one patient the PML diagnosis cannot be confirmed and then he was classified as “possible” PML.

This latter 33-year-old male patient, who did not receive previous immunosuppressant drugs, developed difficulty in sustained attention followed by impaired dexterity on the right hand after 47 infusions. Brain MRI scan revealed a non-enhancing parietal lesion highly suggestive of PML, but the JCV-DNA searching was negative in 5 consecutive CSF samples. The patient refused brain biopsy.

All the patients who were tested were positive for anti-JCV antibodies. Five patients diagnosed before the introduction of STRATIFY test were not tested. No patient was tested anti-JCV negative.

Out of 39 patients, 13 (33.3%) were previously treated with one or more immunosuppressant agents before NTZ beginning. In particular, they were treated with mitoxantrone only (n = 6), azathioprine and mitoxantrone (n = 3); azathioprine only (n = 1), azathioprine and methotrexate (n = 1); cyclophosphamide and mitoxantrone (n = 1); and azathioprine, cyclophosphamide and mitoxantrone (n = 1).

The median ARR decreased from 2 (range 1–4) in the year before NTZ start to 0 (range 0–1) during NTZ treatment (p<0.001).

### PML diagnosis

Patients received a mean (SD) number of 38.6 (18.2) NTZ infusions (range 11 to 86) before PML diagnosis. In one patient (2.6%), the PML diagnosis was made within the first year of NTZ treatment (after 11 infusions), in 9 patients (23.1%) between the 12th and 24th NTZ infusion; in 9 patients (23.1%) between the 25th and 36th NTZ infusion; in 8 patients (20.5%) between the 37th and 48th NTZ infusion; in 7 patients (17.9%) between the 49th and 54th NTZ infusion; in 5 patients (12.8%) after the 64th NTZ infusion.

Patients who were diagnosed before receiving 24 NTZ infusions (n = 10) were older at NTZ start than those who were diagnosed thereafter (n = 29) (43.5±9.7 *vs*. 36.9±8.3 years, p = 0.03); there was no other difference in demographic, clinical, MRI and paraclinical features (including JCV-DNA copies on CSF).

There was no difference in mean number of NTZ infusions received before PML diagnosis between patients previously treated with immunosuppressant drugs (n = 13) and those who did not (n = 26) (35.8±13.4 *vs*. 40.4±20.3 infusions, respectively; p = 0.71).

The PML suspect was made on the basis of deteriorating neurological conditions in 33 (84.6%) cases (symptomatic PML), including cognitive symptoms (n = 13), motor symptoms (n = 7); signs and/or symptoms indicative of brainstem/cerebellar involvement (n = 3); cognitive and motor symptoms (n = 3); motor and brainstem/cerebellar involvement (n = 2); visual deficit (n = 2); epilepsy (n = 1); epilepsy and cognitive symptoms (n = 1); and verbal hallucinations (n = 1). In the remaining 6 (15.4%) cases, there were MRI findings highly suggestive of PML in absence of any detectable change of clinical conditions (asymptomatic PML). These six asymptomatic patients performed MRI every six months (n = 2), every four months (n = 2) or every three months (n = 2).

Interestingly, the diagnosis was quicker for patients who presented only with cognitive symptoms (n = 13) rather than for those with other neurological pictures (n = 20), being the mean days from symptom onset to PML diagnosis of 23.2±11.4 *vs*. 45.7±18.8 days, respectively (p = 0.006).

### MRI patterns and CSF findings

Out of 39 patients, 13 (33.3%) presented with an unilobar lesion, 13 (33.3%) with multilobar lesions, 6 (15.4%) with widespread lesions and 7 (17.9%) with an infratentorial lesion. Contrast-enhancement of PML lesion(s) was found in 14 (35.9%) patients, without any significant relationship with MRI patterns (p = 0.78). In particular, contrast-enhancement occurred in 4 patients who presented with unilobar lesion, in 5 with multilobar lesion, in 2 with widespread lesion and in 3 with infratentorial lesion.

The median number of viral copies/ml detected in CSF at PML diagnosis was 230.5 (range 10–26,300).

Relationships between clinical, MRI features and laboratory findings at PML diagnosis are presented in Table 2. Patients who presented with an unilobar lesion (n = 13) had lower number of viral copies/ml in the CSF compared with patients with other MRI patterns (n = 26), with a statistical trend toward significance (p = 0.09). Interestingly, patients who presented with contrast-enhancement had a lower number of viral copies/ml in the CSF compared with patients without contrast-enhancement (p = 0.03).

**Table 2:** Relationships between MRI features and clinical and laboratory findings at PML onset.

	MRI patterns				Contrast-enhancement	
	Unilobar (n = 13)	Multilobar (n = 13)	Widespread (n = 6)	Infratentorial (n = 7)	Yes (n = 14)	No (n = 25)
Asymptomatic onset (n = 6)	4	0	0	2	1	5
Symptomatic onset (n = 33)	9	13	6	5	13	20
Cognitive symptoms only (n = 13)	2	6	5	0	3	10
Motor symptoms only (n = 7)	4	2	0	1	4	3
Brainstem or cerebellar symptoms only (n = 3)	2	0	0	1	1	2
Cognitive AND motor symptoms (n = 3)	0	2	0	1	2	1
Motor AND brainstem or cerebellar symptoms (n = 2)	0	0	1	1	1	1
Epilepsy (n = 1)	0	0	0	1	1	0
Epilepsy AND cognitive symptoms (n = 1)	1	0	0	0	0	1
Visual loss (n = 2)	0	2	0	0	0	2
Verbal hallucination (n = 1)	0	1	0	0	1	0
JCV-DNA copies/ml on CSF,median (range)	63 (10–26,300)	542 (12–3,932)	327.5 (31–5,174)	294 (15–4,403)	67.5 (21–2,322)	366 (10–26,300)

### Clinical outcome

**Fig 2:**
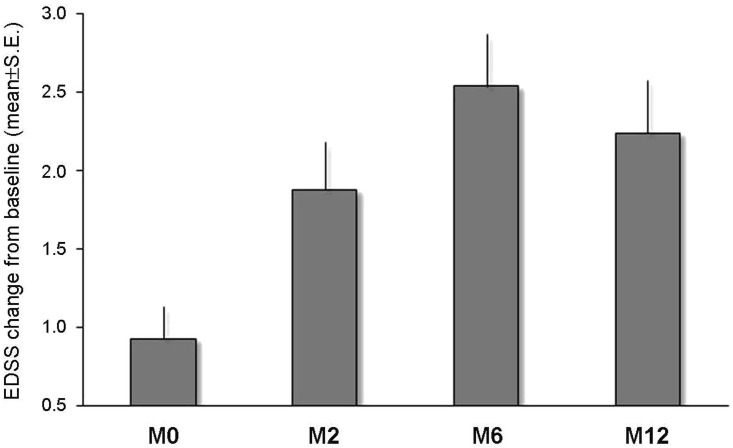
Longitudinal evolution of disability throughout the 12-month observational period.

The mean EDSS score worsened from baseline (i.e. NTZ start) to M0 (i.e. PML diagnosis) (3.9±1.9 *vs*. 4.8±2.1; p<0.001). The assumption of normal distribution of EDSS scores was satisfied at all assessments (p>0.2 by the Kolmogorov-Smirnov test), therefore we analyzed the mean scores throughout the 12-month observational period. The main effect of time on EDSS scores was significant (F_4,148_ = 28.2, p<0.001). The S-N-K post-hoc test revealed that the EDSS score was significantly lower at baseline and M0 than at following assessments (all p-values<0.001). The analysis of EDSS changes from baseline to different following assessments showed that the peak of disability worsening occurred at 6 months from PML diagnosis (Fig 2). At the end of the 12-month observation, 15 (38.4%) patients accumulated up to 1-EDSS point, 9 (23.1%) patients 2-EDSS points, 15 (38.4%) patients 3-EDSS points or more with respect to baseline.

Three out of 39 patients died during the first year after PML diagnosis, resulting in a survival rate of 92.3%; two patients died during PML-IRIS, while one patient died after 6 months from PML diagnosis due to complication of an acute acalculous cholecystitis.

### PML-IRIS

Out of 39 patients, 27 (69.2%) had a PML-IRIS after 82.5±29.2 days following NTZ withdrawal. There was no difference between patients who developed PML-IRIS and those who did not in demographic and clinical characteristics (all p-values>0.15). Out of 12 patients who did not developed PML-IRIS, 9 were treated with plasma exchange (PLEX), and all of them were treated with steroids when their clinical profile was worsening. Nine patients were also treated with an additional therapy (maraviroc, mefloquine or mirtazapine). However, PML-IRIS insurgence was not related to the administration of these therapies, including PLEX, steroids, and other therapies (all p-values>0.12).

Among patients previously treated with immunosuppressant agents (n = 13), 9 developed PML-IRIS and 4 did not (p = 0.87), therefore ruling out the possibility that prior immune suppression might have dampen the PML-IRIS occurrence.

The PML-IRIS emergence was related neither with the number of JCV-DNA copies (median: 172 and 200.5 in patients with or without PML-IRIS, respectively; p = 0.97) nor with the MRI patterns (p-values>0.4). In more details, PML-IRIS occurred in patients who presented with unilobar lesion in 9 cases, multilobar lesions in 9 cases, widespread lesions in 4 cases and brainstem lesion(s) in 5 cases. Accordingly, patients without PML-IRIS presented with unilobar lesion in 4 cases, multilobar lesions in 4 cases, widespread lesions in 2 cases and brainstem lesion(s) in 2 cases.

### Predictors of clinical outcome

We investigate whether the 12-month longitudinal clinical course of patients could have been affected by some factors at PML diagnosis, such as NTZ treatment duration (less or more than 24 infusions), asymptomatic PML, presence of contrast-enhancement at PML suspect, MRI patterns and number of JCV-CSF copies. However, due to the small sample size of subgroups, the results outlined in this paragraph should be should be interpreted with caution and considered only as preliminary.

#### NTZ treatment duration

We found no time by group interaction effect (F_4,148_ = 1.10, p = 0.35) in patients who developed PML before (n = 10) or after (n = 29) receiving 24 NTZ infusions.

#### Asymptomatic PML

**Fig 3:**
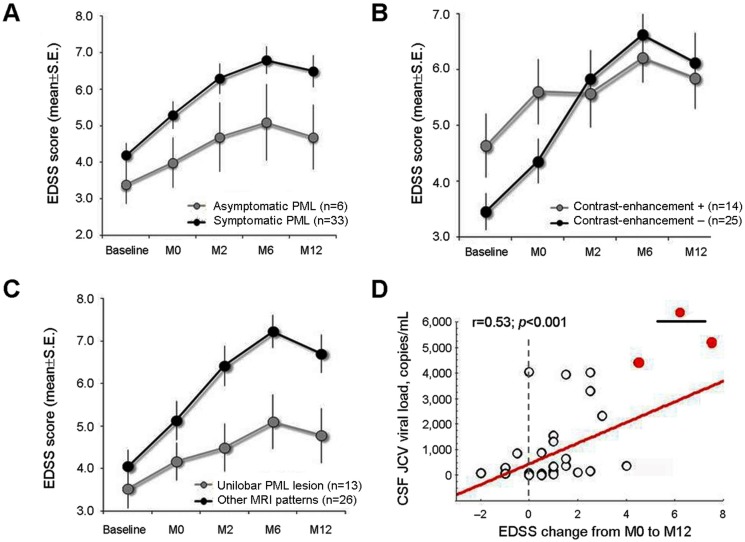
Disability changes throughout the 12-month observational period according to clinical and paraclinical characteristics (A: symptomatic versus asymptomatic presentation; B: absence versus presence of contrast-enhancement at PML diagnosis; C: unilobar versus other MRI patterns; D: correlation with CSF-JCV viral load).

The S-N-K post-hoc test revealed a different longitudinal course in patients with asymptomatic PML (n = 6) and in those with symptomatic PML (n = 33) (Fig 3A). The mean EDSS score of asymptomatic cases did not change from baseline to M0 (3.4±1.2 *vs*. 4.0±1.6, p = 0.18). However, the mean EDSS score worsened from baseline to M2 (4.7±2.3, p = 0.02), M6 (5.1±2.5, p = 0.007) and M12 (4.7±2.1, p = 0.01). We found no difference from M0 to the subsequent assessments and across M2, M6 and M12 assessments. On average, patients with asymptomatic PML accumulated 1.3±1.4 EDSS points from baseline to M12 and 0.7±1.2 points from M0 to M12. In patients with symptomatic PML, the mean EDSS score significantly worsened from baseline to M0 (4.1±1.7 *vs*. 5.1±2.0, p = 0.002), M2 (6.1±2.1, p<0.001), M6 (6.9±2.0, p<0.001) and M12 (6.4±2.3, p<0.001). Moreover, the EDSS score worsened even from M0 to M2, M6, and M12 (p-values<0.01), while there were no differences across the post-PML onset assessments. On average, patients with symptomatic PML accumulated 2.4±2.1 EDSS points from baseline to M12 and 1.3±1.9 points from M0 to M12. All dead patients had a symptomatic PML onset.

#### Contrast-enhancement

We found a significant time by group interaction effect (F_4,148_ = 4.87, p<0.001), indicating a different longitudinal course in patients with (n = 14) and without (n = 25) contrast-enhancement (Fig 3B). The mean EDSS score of patients with contrast-enhancement significantly worsened from baseline (4.6±2.1) to the next assessments (p-values<0.05). There were no differences in EDSS scores from M0 (5.6±2.1) to M2 (5.5±2.2), M6 (6.2±1.6), and M12 (5.8±2.0) (p-values>0.5). Furthermore, there was no difference across different post-PML onset assessments. On average, patients with contrast-enhancement accumulated 1.2±1.3 EDSS points from baseline to M12, and 0.2±1.1 points from M0 to M12. The mean EDSS score of patients without contrast-enhancement significantly worsened from baseline (3.4±1.6) to the following assessments (p-values<0.02). We found significant differences in mean EDSS scores from T0 (4.3±2.0) to M2 (5.8±2.5), M6 (6.7±2.6) and M12 (6.2±2.7) (p-values<0.01). There was no differences across different post-PML onset assessments. On average, patients without contrast-enhancement accumulated 2.8±1.2 EDSS points from baseline to M12 and 1.6±1.1 points from M0 to M12. Dead patients showed no contrast-enhancement at the PML suspect.

#### MRI patterns

We found a time by group interaction effect (F_4,148_ = 2.82, p = 0.02), indicating a different longitudinal course in patients with unilobar lesion (n = 13) and in those who presented other MRI patterns (n = 26) (Fig 3C). The mean EDSS score of patients with unilobar lesion did not change from baseline to M0 (3.5±1.7 *vs*. 4.1±1.6; p = 0.25), while there was a slight worsening at M2 (4.4±1.9; p = 0.09) and an overt worsening at M6 (5.1±2.3, p<0.01) and M12 (4.7±2.3, p<0.01). We found no differences in EDSS scores from M0 to the next assessments (p-values>0.1). On average, patients with an unilobar lesion accumulated 1.2±1.6 EDSS points from baseline to M12 and 0.6±1.7 points from M0 to M12. On the contrary, the EDSS score of patients with MRI patterns other than unilobar lesion worsened from baseline to M0 (4.0±2.0 to 5.1±2.3; p = 0.07) M2 (6.4±2.3), M6 (7.2±1.9) and M12 (6.7±2.2) (p-values<0.001). Significant differences emerged also in EDSS scores between M0 and the following assessment (p-values<0.001). On average, patients with MRI patterns other than unilobar PML lesion accumulated 2.7±2.4 EDSS points from baseline to M12 and 1.6±1.9 points from M0 to M12.

Dead patients had the following MRI patterns: (i) infratentorial lesion; (ii) unilobar lesion located in the left temporal lobe; (iii) widespread pattern involving the fronto-temporo-parietal white matter of the left hemisphere.

#### Viral load

We found the number of viral copies to be strongly correlated with EDSS changes: M2-M0 (r = 0.47; p = 0.002), M6-M0 (r = 0.49; p = 0.001), and M12-M0 (r = 0.53; p<0.001) (Fig 3D). The highest values of viral copies (>4,403) were detected in the 3 patients who died within the first year after PML diagnosis.

## Discussion

The current paper analyzes the longitudinal data of 39 Italian patients who developed NTZ-related PML between 2009 and 2016. Our analysis largely confirms literature data and offers some new insights about this topic.

Consistently with previous observations, NTZ-related PML evolves in a timeframe from 6 to 12 months, with the peak of disability at 6 months and a variable degree of recovery at 12 months from the diagnosis [14,17,18,37,38]. Accordingly, we observed all deaths around 6 months from PML diagnosis [14,18]. Our study corroborates previous findings that asymptomatic PML, more localized brain involvement and lower JCV viral load at PML diagnosis are associated with better outcomes. The three deaths occurred indeed in patients with symptomatic PML and higher numbers of CSF-DNA viral copies at PML diagnosis. This is in line with previously published data on predictors of survival and functional outcomes [16–18] and indicates that the later the PML diagnosis was made, the more the disease was widespread. Therefore, our study strongly supports the deferral of further NTZ dosing until exclusion of PML diagnosis in case of unexpected clinical worsening or new MRI lesions occurring beyond the first year of therapy [39].

The current paper also highlights some new features with respect to existing literature, including: (i) a higher survival rate; (ii) a higher percentage of asymptomatic patients at PML suspect; (iii) a shorter time to diagnosis in patients with a cognitive PML onset; (iv) a relevant proportion (25.6%) of patients who developed NTZ-related PML before the 24th infusion; (v) a different clinical course between patients with and without MRI enhancement of PML lesion(s); (vi) a lower prevalence of PML-IRIS onset.

The 92.3% survival rate after one year from PML diagnosis found in the Italian cohort is higher than most of previously published studies (70–80%) [1,18,37]. This latter finding probably reflects the higher proportion (15.4%) of asymptomatic PML in our cohort compared with other datasets (see for example the paper by Dong-Si and coll. [17] reporting an 8% of asymptomatic PML). As reported by previous studies, asymptomatic PML cases showed a single localized lesion at brain MRI, with unilobar or infratentorial patterns [39]. The presence of more localized lesions in asymptomatic patients probably mirrors the stringent application of national recommendation regarding more frequent MRI scans in patients at high risk of developing PML [24]. In our cohort, asymptomatic PML cases underwent brain MRI scans two, three of four times per year, supporting the advice that more frequent MRI examinations are helpful for an early identification of PML. This is also in line with the recently published international MAGNIMS consensus guidelines [30], which recommended indeed to monitor every 3–4 months with brain MRI screening all NTZ-treated patients who are at high risk for developing PML.

Among the 33 symptomatic PML cases, we observed a shorter time to diagnosis in patients who presented with cognitive symptoms than in those who presented with other symptoms. We may speculate that cognitive and behavioural changes rather than emerging or deteriorating motor symptoms alerted clinicians to the possible PML diagnosis. As for new MRI lesions, there is also recommendation that any new symptom or occurring beyond the first year of treatment should be treated with suspicion [6,24,40,41]. However, fluctuations of motor function, which are frequent in patients with MS and sometimes are misinterpreted as MS relapses, could have delayed the correct diagnosis [42]. Moreover, it has been reported that alternative diagnoses are considered before PML in nearly two-thirds of patients, and that more than three-quarters of PML patients suffered from diagnostic delay, irrespective of their underlying immunosuppressive condition [43].

In our cohort, the PML diagnosis was made before the 24th months of treatment in 10 (25.6%) patients. This finding raises some concerns about the currently recommended risk management plan that considered at higher risk for PML those patients treated with NTZ for more than 24 months [11]. Out of 638 confirmed cases of NTZ-related PML reported at March 2016, about 15% have been indeed diagnosed before two years of treatment, according to manufacturer data [10]. Our data suggest that an older age at NTZ start might be a risk factor for developing PML before 24 infusions. Interestingly, advanced age has been reported to be also a risk factor for PML under other MS therapies, such as fingolimod and dimethyl fumarate [44], suggesting that age may represents an additional risk stratifier for PML in patients treated with MS therapeutics.

The 35.9% of patients who presented with MRI enhancement at PML diagnosis is consistent with recent literature that reported enhancement in 41% of cases [35]. More interestingly, our data suggest a different clinical course between patients with and without enhancement at PML diagnosis. In contrast with a previous observation [13], patients without contrast enhancement experienced a worse longitudinal course than those with MRI enhancement. This is also supported by the fact that none of the dead patients showed MRI enhancement. Contrast-enhancement indicates an inflammatory response that often occurs in the setting of immune restoration and may represent the first attempt to get rid of JCV [36]. Therefore, in the absence of specific treatments against the JCV brain infection, restoration of immune system should be considered pivotal for survival and clinical recovery and should be not impeded by steroids, unless PML-IRIS occurs. Although we are aware that this latter hypothesis should be considered with caution, our results deserve attention and future confirmation. Previous literature data suggested indeed that MRI enhancement of PML lesions are generally associated with a favourable prognosis [36].

Finally, we found a 69.2% prevalence of PML-IRIS, in contrast with current literature stating that almost all patients developed PML-IRIS [17,18]. This lower PML-IRIS prevalence seems to be not related to any demographic and clinical data, administration of PLEX, steroids, maraviroc, mefloquine or mirtazapine. However, we cannot exclude that the widely heterogeneous use of steroids (different dosages, administration route and schedules) and PLEX (number of cycles) could have affected the PML-IRIS occurrence. An alternative hypothesis encompasses a possible underestimation of patients with PML-IRIS in our cohort, since we did not include in this subgroup those patients with early signs of PML-IRIS, as recently described [19].

This study has several limitations and therefore its findings should be interpreted cautiously.

Firstly, the current data are derived from an observational retrospective analysis. This aspect, together with a relatively small sample size (PML is an uncommon adverse event), makes difficult to overcome the exploratory nature of the study.

Secondly, as main outcome we adopted the pseudo-ordinal EDSS score, whose psychometric properties could have partially affected our findings [45]. However, EDSS was consistently and reliably used across Italian sites to assess the disability evolution before and after the PML diagnosis, thus allowing us to obtain a consistent and valid longitudinal measure of disability for all patients.

Thirdly, mixed treatments were adopted to treat PML and PML-IRIS, thus missing the chance to provide any indication about which pharmacological (e.g. mefloquine, mirtazapine, maraviroc, PLEX, etc.) and/or non-pharmacological approach that may be potentially useful to improve the survival rate and final outcome.

Lastly, data on anti-JCV antibodies index values are lacking, being available only since 2013 [12].

On the other hand, our study has also number of strengths, including an homogeneous observational period length, full data availability and data harmonization which led to an uniform definition of PML-IRIS.

In conclusion, our analysis on Italian PML cases supports the utility of frequent MRI examination in patients treated with NTZ and confirms that early diagnosis can prevent a widespread CNS involvement, leading to a more limited disease (i.e. pre-clinical presentation, unilobar lesion, low viral load) associated with a better outcome and higher survival rate. However, despite such encouraging observation, a relevant proportion of patients who survive PML had substantial and permanent disability, thus requiring additional efforts to explore potential strategies to mitigate the PML-related damage.

## Supporting Information

S1 File STROBE checklist. (DOC)

S1 Table Dataset reporting data from individual PML cases collected by the Italian PML registry. (XLSX)
